# Sheet-based extrusion bioprinting: a new multi-material paradigm providing mid-extrusion micropatterning control for microvascular applications

**DOI:** 10.1088/1758-5090/ad30c8

**Published:** 2024-03-14

**Authors:** Ryan Hooper, Caleb Cummings, Anna Beck, Javier Vazquez-Armendariz, Ciro Rodriguez, David Dean

**Affiliations:** 1 Department of Biomedical Engineering, The Ohio State University, Columbus, OH 43210, United States of America; 2 Department of Biology, The Ohio State University, Columbus, OH 43210, United States of America; 3 Department of Biochemistry, The Ohio State University, Columbus, OH 43210, United States of America; 4 Department of Materials Science & Engineering, The Ohio State University, Columbus, OH 43210, United States of America; 5 Escuela de Ingeniería y Ciencias, Tecnológico de Monterrey, Monterrey 64849, NL, Mexico; 6 Laboratorio Nacional de Manufactura Aditiva y Digital (MADiT), Apodaca 66629, NL, Mexico; 7 Departamento de Ingeniería Mecánica y Materiales Avanzados, Escuela de Ingeniería y Ciencias, Tecnológico de Monterrey, Monterrey 64849, NL, Mexico; 8 Department of Plastic and Reconstructive Surgery, The Ohio State University, Columbus, OH 43210, United States of America

**Keywords:** sheet-based 3D bioprinting, multi-material, microvasculature, perfusion, chaotic printing, melt electrowriting

## Abstract

As bioprinting advances into clinical relevance with patient-specific tissue and organ constructs, it must be capable of multi-material fabrication at high resolutions to accurately mimick the complex tissue structures found in the body. One of the most fundamental structures to regenerative medicine is microvasculature. Its continuous hierarchical branching vessel networks bridge surgically manipulatable arteries (∼1–6 mm) to capillary beds (∼10 *µ*m). Microvascular perfusion must be established quickly for autologous, allogeneic, or tissue engineered grafts to survive implantation and heal in place. However, traditional syringe-based bioprinting techniques have struggled to produce perfusable constructs with hierarchical branching at the resolution of the arterioles (∼100-10 *µ*m) found in microvascular tissues. This study introduces the novel CEVIC bioprinting device (i.e. Continuously Extruded Variable Internal Channeling), a multi-material technology that breaks the current extrusion-based bioprinting paradigm of pushing cell-laden hydrogels through a nozzle as filaments, instead, in the version explored here, extruding thin, wide cell-laden hydrogel sheets. The CEVIC device adapts the chaotic printing approach to control the width and number of microchannels within the construct as it is extruded (i.e. on-the-fly). Utilizing novel flow valve designs, this strategy can produce continuous gradients varying geometry and materials across the construct and hierarchical branching channels with average widths ranging from 621.5 ± 42.92% *µ*m to 11.67 ± 14.99% *µ*m, respectively, encompassing the resolution range of microvascular vessels. These constructs can also include fugitive/sacrificial ink that vacates to leave demonstrably perfusable channels. In a proof-of-concept experiment, a co-culture of two microvascular cell types, endothelial cells and pericytes, sustained over 90% viability throughout 1 week in microchannels within CEVIC-produced gelatin methacryloyl-sodium alginate hydrogel constructs. These results justify further exploration of generating CEVIC-bioprinted microvasculature, such as pre-culturing and implantation studies.

## 1. Introduction

### 1.1. The vascularization problem

Millions of people need tissue and organ transplants every year, a clinical concern further complicated by significant donor shortages worldwide [1, 2]. To supplement naturally derived donor tissues (i.e. autografts, allografts, and xenografts), various biofabrication methods are currently in development [3]. One notable biofabrication method is bioprinting, which involves 3D printing pre-determined geometries using viable cells, biomaterials, and biomolecules as printing materials [4]. Bioprinted tissue constructs, which already serve as modeling tools for drug screening and research, could generate precisely customizable alternatives to the millions of naturally derived tissue grafts implanted each year [5].

For bioprinting therapies to reach the clinic, however, they must include sufficient and sustained vascularization upon implantation [6]. In almost all bodily tissues, cells must be within 200 *µ*m of a nearby capillary to allow sufficient nutrient delivery and waste removal for long-term survival [7]. This is a big reason why autologous free flap grafting is commonly combined with bone or other grafted tissue during reconstructive surgical procedures. With this technique, autologous tissue can be sectioned and transferred to a new bodily region while retaining functional vasculature, either within the primary tissue or quickly provided by highly microvascular secondary tissue (e.g. muscle or fat). This allows for immediate blood perfusion upon implantation through microsurgical anastomosis with adjacent blood vessels. However, autologous free flap grafting inherently leads to considerable comorbidity and often pain at the donor site [8].

Various strategies have been explored to provide or initiate the vascularization of tissue constructs produced via bioprinting and other tissue engineering methods. These have traditionally involved methods such as growth factor delivery, cell co-culture, and mechanical stimulation to induce spontaneous capillary bed development [9]. While demonstrating the incredible malleability of endothelial cells (ECs), spontaneous organization does not ensure consistent blood supply within the 200 *µ*m diffusion limit across a whole construct, nor clear locations for surgical connection and integration with adjacent host small-diameter vessels [10]. Essentially, missing from the equation is microvasculature’s natural function to hierarchically branch from small-diameter vessel-scale (∼1–6 mm) to capillary-scale (∼10 *µ*m), reducing blood pressure with each branch division and consistently distributing blood supply throughout capillaries less than 200 *µ*m apart [11].

Thus, the vascularization tissue engineering focus has shifted toward creating hollow, hierarchical branching networks that resemble the natural organization of the vascular tree. In addition to bioprinting, many novel fabrication methods are being applied to this pursuit, such as electrospinning, casting, and molding [12–14]. While all of these techniques have potential, they typically involve dense, impenetrable biomaterials. Laser-assisted direct writing can produce microchannels in cell permissive hydrogels for continued vascular remodeling beyond the initially produced network, but the associated laser exposure can negatively impact cell viability and structural integrity [15]. On the other hand, bioprinting strategies can produce hollow, cell-seeded hydrogel channels without requiring laser exposure, but to this point have struggled to successfully mimick the entire resolution range of natural microvascular networks [16]. Thus, the inability to fabricate an artificial microvascular graft with bioprinting is a technology gap that, if a useful technology emerged, would be a paradigmatic shift in the field of biofabrication [16–19].

### 1.2. Pursuing clinical bioprinting technologies

To accurately replicate microvasculature and other complex tissues, bioprinting will likely need to create layers as thin as a single cell [20, 21]. Extrusion bioprinting (i.e. driving cell-laden liquid hydrogel through a nozzle to be solidified upon deposition) is the most well researched and relatively low cost method that could provide scalable tissue and organ bioprinting. However, the cell-laden hydrogel filaments resulting from traditional extrusion bioprinting have the lowest resolution of all bioprinting modalities (∼100 *µ*m). Light-assisted bioprinting (i.e. inducing hydrogel crosslinking via laser or projected image) reaches significantly higher resolutions (<10 *µ*m) but often requires significant ultraviolet (UV) light exposure, introducing well-documented risk of DNA damage and cancer [22]. Some light-assisted bioprinting techniques utilize visible light to address these concerns, however this requires stronger light sources to provide the same print times and curing quality as UV-based methods [23]. Additionally, researchers of both UV and visible light bioprinting technologies are challenged with developing well-performing photoinitiators that are non-toxic in cell-laden hydrogel formulations [24].

In addition to achieving high resolutions, bioprinting must also incorporate multiple materials and cell types to replicate the highly structured spatial patterning of different cell types found in native, functional tissues [25]. In microvascular tissues, for example, vessel walls are primarily comprised of ECs surrounded by a basement membrane interspersed with pericytes (PCs) as vessels branch from arterioles into capillaries and post-capillary venules. Among the various functions performed by these cells, ECs form the inner walls of new vessels (i.e. vasculogenesis, primarily during development) and sprout into new vessel branches (i.e. angiogenesis, during development and vascularization of hypoxic tissues or grafts) [26]. PCs help regulate these processes, provide structural support, and modulate cell and biochemical transport in and out of vessels [27]. Bioprinting constructs containing both ECs and PCs, while still a simplification, can mimic the multi-faceted interactions between these cell types in natural microvascular tissues [16].

Multi-material bioprinting has thus been explored in the interests of replicating the complexities of natural tissues. Extrusion bioprinting of cell-laden hydrogel filaments is the most commonly researched multi-material bioprinting strategy, with both single-nozzle and multi-nozzle devices being developed [28, 29]. While multi-nozzle devices avoid material cross-contamination, using a single nozzle allows for one to switch material without stopping the extrusion process, an event that impacts the construct’s structural integrity and adds additional nozzle alignment and deposition challenges for the device [30]. Multi-material bioprinting has also been applied to handheld devices that allow for efficient, *in situ* deposition of fragile tissue constructs directly onto wound sites [25, 31].

### 1.3. Chaotic printing

Chaotic printing is a patent-pending, multi-material extrusion bioprinting strategy that utilizes chaotic advection in a kenics static mixer (KSM) to produce channels within a filament, creating structures with significantly higher resolution and surface area-to-volume ratios than is possible with traditional extrusion bioprinting devices [32]. By mixing two ink inputs into alternating adjacent channels within a filament, chaotic printing can produce channels under 10 *µ*m in width without requiring a smaller nozzle than traditional extrusion bioprinters use, avoiding clogging and compromised cell viability from increased shear stresses [33]. Chaotic printing reaches the higher resolutions found in light-assisted bioprinting modalities without the aforementioned toxicity concerns of UV light and photoinitiator hydrogel components. Chaotic printing has been demonstrated as a viable strategy for inducing cell alignment, providing vacant channels for pre-vascularization and rapid cell expansion in high surface-area-to-volume bioreactor systems [24–36]. Chaotic printing has also recently been explored for producing radial and axial micropatterns with up to 8 inks at once [37].

### 1.4. The CEVIC device

**Figure 1.**
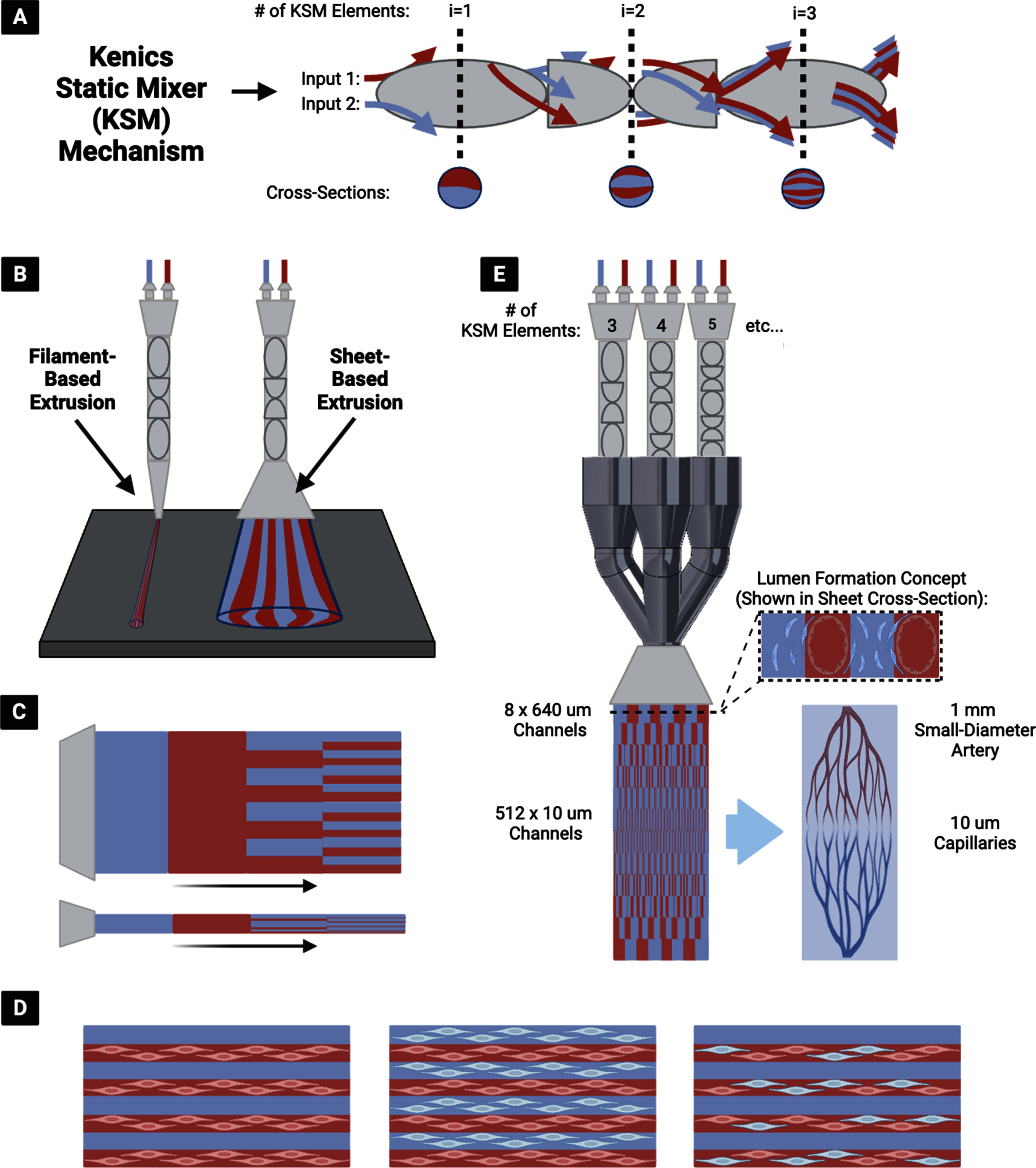
(A) The kenics static mixer (KSM) mechanism employed in chaotic printing, utilizing chaotic advection to mix two or more (shown as two) fluid inputs into alternating striations. Each KSM ‘element’ (i.e. helicoidal blade that divides and rotates the incoming fluid) included in sequence doubles the number of striations produced. (B) (Left) Original chaotic printing concept for extrusion of filaments containing striations (i.e. alternating internal channels) of two or more hydrogels, vs. (Right) newly introduced concept of fanning the output into a wide sheet while maintaining the alternating internal channel structure—producing a workable micropatterned construct in one extrusion. (C) CEVIC (Continuously Extruded Variable Internal Channeling) device can provide any combination of bioink/channel switching mid-extrusion through a single printhead. (D) Microchannels can house one or more cell types in multiple combinations to promote cell alignment, heterocellular interactions, and/or perfusion, depending on the desired application. (E) The CEVIC device printhead, combining multiple flow pathways through varying numbers of KSM elements in sequence, to produce hydrogel sheets with continuous hierarchical branching channels from artery to capillary-scale (left), a design concept currently being explored as a potential template for culturing microvascular tissue constructs (right).

The CEVIC (Continuously Extruded Variable Internal Channeling) device is a novel, patent-pending invention that uses chaotic printing principles to achieve novel microvascular patterns (figure 1). The device extrudes hydrogel sheets instead of filaments while maintaining the alternating adjacent channeling structure unique to chaotic printing (figure 1(A)). These channels have promise as both cell-seeded microvascular structures or for ‘fugitive’/‘sacrificial’ inks that set up vacated spaces for nutrient inflow, waste product removal, or subsequent cell seeding. The CEVIC device allows a complete micropatterned construct covering a relatively large area (e.g. at least 25–300 mm^2^) to be produced in one extrusion, rather than requiring many passes of filament deposition. Additionally, the CEVIC device can switch between materials/bioinks and kenics mixers mid-extrusion to create variations in bioink type, as well as channel number and thickness throughout a continuous sheet or filament extruded from a single printhead (figure 1(B)). These channels can provide a basis for inducing cell alignment [35], positioning two or more cell types in adjacent striations, and allowing perfusion of blood or nutrient media through the tissue construct (figure 1(C)). This capability also allows the device to produce hierarchical branching of internal channels with widths spanning from artery diameter-scale to capillary-scale, creating potential for complex, heterocellular microvascular construct fabrication (figure 1(D)). The CEVIC device can be used for both handheld and fully automated stage bioprinting.

To our knowledge, the CEVIC device is the first example of sheet-based extrusion bioprinting for applications outside of *in situ* bioprinting for wound repair [31]. Additionally, it is the first time chaotic printing has been translated to sheet-based extrusion bioprinting, rather than filament deposition, while being the first attempt at modulating channel number and width mid-extrusion throughout a chaotically printed construct for the purposes of replicating the hierarchical branching pattern of native microvasculature with accurate dimensions.

This study verifies the intended design and function of the CEVIC device while providing a proof-of-concept *in vitro* multi-cellular experiment to demonstrate the device’s potential for producing implantable microvascular tissue constructs.

## 2. Materials and methods

### 2.1. KSM printheads, static valve, and perfusion chamber creation

**Figure 2.**
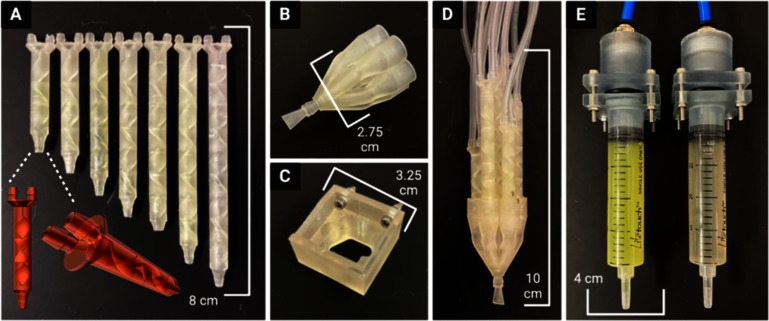
All 3D printed device components (A) Kenics static mixers (KSMs) with 3-9 mixing elements to produce 8, 16, 32, 64, 128, 256, and 512 channels, respectively. Frontal and isometric views of a KSM *.STL file show the internal mixing elements. A 7-input static valve (B) is either controlled by hand or fixed to a 3D printer (C). (D) KSMs are fit into the 7-input static valve to direct the selected flow pattern through a fanning nozzle outlet, producing a hydrogel sheet. (E) Components to maintain air-tight seal between air pump and syringes containing hydrogel inks.

A kenics static mixing (KSM) printhead *.STL file utilized in previous chaotic printing work [32, 34–36] was edited with Autodesk Meshmixer (San Francisco, CA, USA) computer-aided-design (CAD) software. The three mixing elements contained in the original printhead that divide two inputs into 8 layers were duplicated and transformed to construct six new KSM designs with four to nine mixing elements (for outputting 16, 32, 64, 128, 256, and 512 channels, respectively, within the resulting hydrogel sheet) (figure 2(A)).

A novel 7-input static valve printhead (figure 2(B)) and printhead holder piece (figure 2(C)) were designed using SolidWorks CAD software (Waltham, MA, USA). Each input to the printhead was modeled to fit one of the seven KSMs (figure 2(D)). Components were also designed to couple the pneumatic air source to the hydrogel/bioink syringes (figure 2(E)). A fan-shaped (i.e. oblong, 5 mm width by 0.5 mm height) nozzle design was combined to the output of the static valve printhead in MeshMixer CAD software from Autodesk (San Francisco, CA, USA).

All parts were exported as *.STL files and imported into EnvisionTec (Dearborn, MI, USA) Perfactory RP software. This software positioned the *.STL files on the buildplate, built supports, and produced job files for use on a Perfactory P3 DLP 3D printer. Job files were uploaded to the printer using Perfactory Observer software and the parts were printed with E-Shell 300 and E-Clear resins from EnvisionTec. After printing, each part was washed with a sequence of isopropanol, acetone, ethanol, and deionized (DI) water before drying in a dessicator. Parts were then post-cured for 40 min in a 3D Systems (Rock Hill, SC, USA) Procure 350 UV light box.

### 2.2. Device set-up

**Figure 3.**
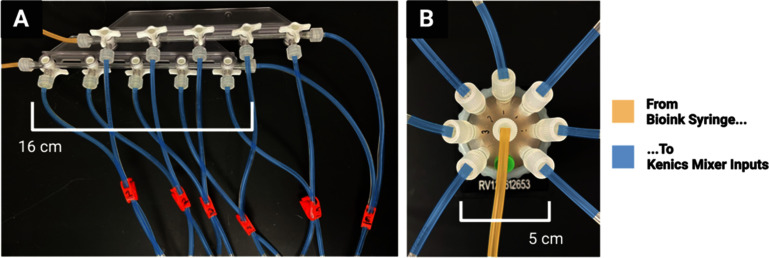
Two options for mechanical valves to select channel number and width. (A) Manual option, valves are turned by hand to direct two hydrogel inks into a desired KSM to produce the intended number and width of channels in the outputted construct. (B) Automatic alternative using an electric rotary valve. Valve rotates on a stepper motor to direct a hydrogel ink into the desired kenics mixer.

Two 20 ml syringes were filled with hydrogels of choice (i.e. depending on test of interest) slightly past 20 ml, tapped vertically to dislodge any bubbles, and pushed to the 20 ml to remove the bubbles. Each syringe is then connected to the inputs of either two manual channel valves (figure 3(A)) (for manual mixer selection) or two electric rotary valves from Aurora Pro Scientific (Midland Park, NJ, USA) (figure 3(B)) (for automated mixer selection) using two 1/16″ ID rubber tubing of equal length. Next, enough 1/16″ tubing of equal length were cut to connect each valve output to one of the two inputs on a respective KSM, so that each mixer has two different inputs (e.g. 14 tubes for seven KSMs). The syringes in the valve-syringe apparatus were then secured with the coupling components to a MAC100Q air compressor (i.e. pump) (Makita, Anojo, JP).

The seven KSMs were each fitted with a 1/4″ long piece of 1/8″ ID rubber tubing at their outlet to form a water-tight seal in the seven-input static valve printhead. They were then inserted securely into the static valve printhead so that the inlets of the 3, 5, 7, and 9-element KSMs were parallel to the extrusion plane of the fanning nozzle outlet, while the 4, 6, and 8-element KSMs were inserted with their inlets perpendicular to the extrusion plane of fanning outlet nozzle. This arrangement allowed all channels to be extruded on the same plane despite the sequential 90° axial rotation of each mixing element within the KSMs.

### 2.3. Device operation

#### 2.3.1. Hydrogel printing

**Figure 4.**
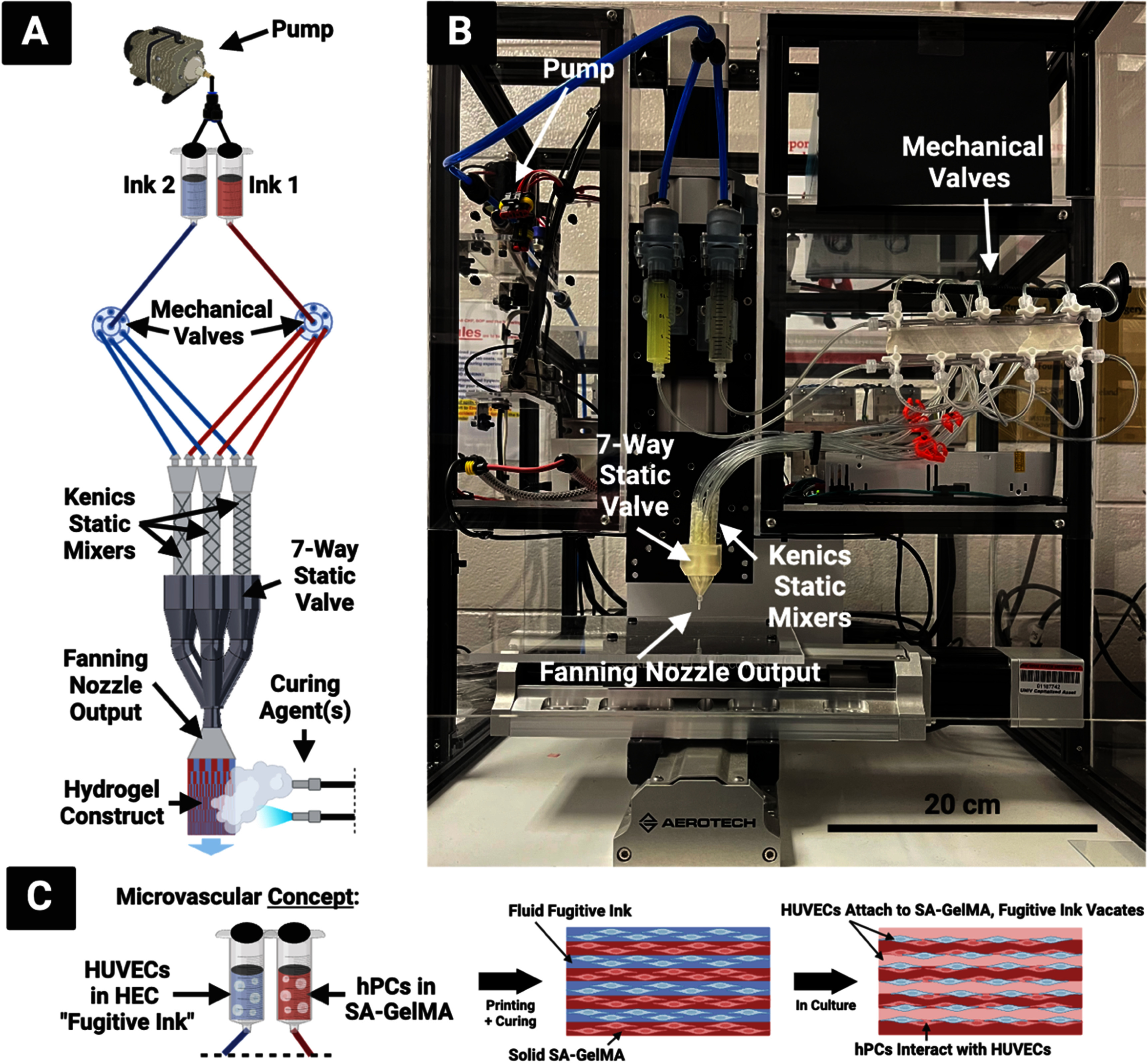
(A) CEVIC device schematic, portraying electric rotary valves for automated input selection. Mechanical valves control which kenics static mixer receives the two ink inputs. The chosen mixer creates a certain number of alternating channels of the two inks within the volume that exits the nozzle output to form a sheet, which is then solidified via curing agent(s). Because varying the number of kenics mixer elements varies the channel number and width, controlling the mechanical valves therefore controls channel number and width in the resulting sheet construct mid-extrusion. (B) Current device iteration, with non-electric/manual mechanical valves for manual input selection. (C) CEVIC microvascular tissue fabrication concept that is first explored in this study with an endothelial cell (HUVEC) and pericyte (hPC) proof-of-concept experiment.

The pump is turned on to pass the hydrogels through both syringes at a 1 ml min^−1^ flow rate, with 0.5 bar supplied to each syringe, to first fully saturate the apparatus. Each KSM is checked visually for air bubbles, which are removed by tilting the seven-input static valve printhead upward while hydrogel is flowing through. Once the whole apparatus is saturated with hydrogel, the printhead is fit into the holder piece on a robotically-controlled 3D printing apparatus developed in previous work in our lab [38] and controlled by LabVIEW from National Instruments (Austin, TX, USA) (figure 4).

The pump is then started again while the basement is simultaneously moving at a constant 3.3 mm s^−1^, a speed calculated to match the 1 ml min^−1^ flow rate and thereby produce uniformly wide sheets with consistent channels. For handheld printing, the basement was kept in place while the printhead was guided by hand. During manual mixer selection, the stopcocks on the manual valves were manually switched closed on each outlet at uniform intervals in order to direct the flow of hydrogel from one kenics mixer to another midflow. For automated mixer selection, two rotary valves can be programmed via LabVIEW (or other software) to switch after a designated period of time between corresponding tubes leading to each kenics mixer.

#### 2.3.2. Post-print curing

Immediately after extrusion, the channeled hydrogel sheet can be cured/solidified. With UV-sensitive hydrogels, such as gelatin methacryloyl (GelMA), the sheets are exposed to 365 nm UV light from an Omnicure S2000 (Laude, GR, NL) for 30 s. While UV exposure can negatively affect cell viability, as mentioned in section 1.2, previous studies on chaotic printing have shown the chosen wavelength and duration to be sufficient for solidifying GelMA throughout hydrogel constructs without a detectable effect on cell viability [34, 36]. For hydrogels that physically crosslink in the presence of calcium chloride (CaCl_2_), such as sodium alginate (SA), the sheets are sprayed with a fine mist of 4% (wt./vol.) CaCl_2_ using either a handheld sprayer bottle or an ultrasonic atomizer. Applying CaCl_2_ in the form of gradually increased misting intensity allows the gel to be crosslinked with minimal impact on physical gel structure, as previously demonstrated [39, 40]. After waiting approximately 30 s, CaCl_2_ droplets are then added to the sheet with a Pasteur pipet to completely soak the sheet. At least 5 additional minutes are then allowed to ensure complete crosslinking before handling the sheets. If the hydrogel contains both GelMA and SA, the UV and CaCl_2_ crosslinking steps are completed in succession.

#### 2.3.3. Combining with MEW biotextile scaffolds

The hydrogel sheet constructs produced with this device can be combined with woven thermoplastic polymer fiber scaffolds produced by melt electrowriting (MEW) to combine properties of both materials. This is accomplished simply by extruding directly over a MEW scaffold before conducting the post-print curing steps described in section 2.3.2. This allows hydrogel to crosslink around the MEW scaffold, effectively fusing both together. The 500 *µ*m polycaprolactone (PCL) scaffolds were produced with a MEW device using previously defined biotextile fabrication methods [38].

### 2.4. Construct fabrication testing

#### 2.4.1. Hydrogel preparation

SA was added to Dulbecco’s phosphate-buffered saline (DBPS) from Gibco (Billings, MT, USA) at 4% (wt./vol.) and mixed at 70 °C until fully dissolved. Three to five drops of FluoSpheres polystyrene microspheres from Invitrogen (Waltham, MA, USA) were added to half of the prepared 4% SA, while the other half was kept clear. The final solutions were maintained at 37 °C until use. To create a ‘fugitive ink’ (a.k.a. ‘sacrificial ink’), as developed in previous chaotic printing work [35, 36], a 0.8% (wt./vol.) solution of hydroxyethyl cellulose (HEC) was prepared. HEC was mixed in DI water at 80 °C until fully dissolved, then maintained at 37 °C until use.

#### 2.4.2. Channel visualization and measurement

For visualization purposes, 4% SA with fluorescent microspheres and 4% SA without microspheres were used as the two hydrogel inputs in the device to produce hydrogel sheet constructs with alternating clear and fluorescent channels. A 365 nm UV light was shined on the resulting hydrogel sheets to illuminate the fluorescent layers while pictures were taken with a Plugable USB Digital Microscope (Redmond, WA, USA). ImageJ software (NIH) was used to measure the width and thickness of the hydrogel sheet constructs, as well as channel widths, with six replicates for each respective measurement.

#### 2.4.3. Perfusion testing

To create hydrogel sheet constructs with vacant inner channels, 4% SA with fluorescent microspheres and 0.8% HEC were used as the two hydrogel inputs in the device. During the CaCl_2_ crosslinking process described in section 2.3.2, SA solidified while HEC remained fluid as a fugitive ink. Resulting constructs were soaked in DI water overnight to allow the HEC to diffuse out, leaving behind vacant internal channels. A 1 ml insulin syringe was then used to inject vacant channels with orange food-colored DI water to demonstrate channel perfusion.

### 2.5. Cell viability testing

#### 2.5.1. Sterile hydrogel preparation

A 3% (wt./vol.) GelMA, 2% SA, and 0.1% lithium phenyl-2,4,6-trimethylbenzoylphosphinate (LAP) solution was prepared in DPBS to serve as a base for the cell-laden bioink. GelMA was chosen for its cell adhesion-promoting properties, while SA serves as structural support for the resulting solidified gel, with minimal added cell interactions [34]. LAP powder was first constituted in DPBS and passed through a 0.22 *µ*m filter via syringe for sterilization. Lyophilized GelMA and SA powder were exposed to UV-C light for 15 min before being added to the LAP solution in a sterile biosafety cabinet. The solution was mixed in sterile conditions at 70 °C until fully dissolved, then maintained at 37 °C until use. To produce a sterile 0.4% HEC solution, HEC powder was similarly exposed to UV-C light for 15 min before being added to sterile DI water. The HEC solution was mixed 80 °C until fully dissolved, then maintained at 37 °C until use.

#### 2.5.2. Cell preparation

EndoGRO human umbilical vein endothelial cells (HUVECs) and human brain vascular pericytes (hPCs) were obtained from Millipore Sigma (Burlington, MA, USA) and iXCells Biotechnologies (San Diego, CA, USA) at passage 1 and 2, respectively. Each cell type was thawed, seeded in two T175 flasks at 500 000 cells per flask, and incubated at 37 °C and 5% CO_2_. HUVECs were supplied with EBM Endothelial Cell Growth Basal Medium and SingleQuots Supplements and Growth Factors from Lonza (Walkersville, MD, USA), while hPCS received Human Pericyte Growth Medium 5 from iXCells Biotechnologies. Media changes occurred every 72 h. Once the flasks reached approximately 80%–90% confluence, the cells were washed three times with phosphate-buffered saline (PBS) (Gibco) and detached with TrypLE (Gibco). The resulting cell suspensions were pelleted down by centrifuging at 1200 rpm for 10 min and then aspirating the remaining media. Cells were then reconstituted in fresh media. The 10 *µ*l of the cell suspension was combined with 80 *µ*l of PBS and 10 *µ*l of TrypanBlue (Gibco) before being counted with a hemocytometer.

Based on the measured cell densities, the suspensions were transferred to new centrifuge tubes so that there were 20 million cells in each tube. Media was aspirated and 10 ml of 3% GelMA 2% SA 0.1% LAP hydrogel was added to the hPC pellet, while 0.4% HEC was added to the HUVEC pellet. These solutions were aspirated up and down to homogenize the cells within the gels, each at a density of 2 million cells ml^−1^. The pipet tips used at this step were trimmed approximately 1/3rd the distance from the tip to facilitate transfer of the relatively viscous hydrogel and minimize shear stress on the cells.

#### 2.5.3. Bioprinting

The 3% (wt./vol.) GelMA 2% SA 0.1% LAP with hPCs and 0.4% HEC with HUVECs were used as the two hydrogel inputs in the device for the experimental group, while 3% GelMA 2% SA 0.1% LAP without cells and 0.4% HEC with HUVECs were used for the control group. The HUVEC-laden HEC ink was utilized to attempt a new ‘endothelial seeding’ method, whereas HUVECs could attach around the edges of each vacant channel as the fugitive ink vacates the construct. Sterile bioprinting was conducted by manually dragging the printhead across a well plate lid within a biosafety cabinet. Printhead components were sterilized with autoclaving and ethanol soaks, where appropriate. The kenics mixer with three mixing elements was chosen to create cell-laden hydrogel sheet constructs with approximately 8 alternating channels (i.e. 4 channels of hPC-laden gel alternating with 4 vacant channels lined with HUVECs). The constructs were cured within the biosafety cabinet using the UV and CaCl_2_ methods described in section 2.3.2. To prevent CaCl_2_ mist from entering the biosafety cabinet air flow, it was sprayed over the constructs within a folded enclosure of sterile aluminum foil. Printed constructs were cut into 1 cm segments with a razor blade before being placed in 24-well plates containing a 50:50 combination of the respective endothelial and PC cell growth media formulations, respectively. The well plates were incubated at 37 °C and 5% CO_2_ for 1 week, with media changes every 72 h.

#### 2.5.4. Cell viability and proliferation

A Live/Dead fluorescent viability assay from Invitrogen (Waltham, MA, USA) was used to determine cell viability percentage in each sample across the experimental and control groups. On days 1 and 7, triplicates from each group were washed three times with DPBS before being submerged in 2 *µ*M and 4 *µ*M of calcein AM and 4 *µ*M ethidium homodimer-1, respectively, in DPBS for 45 min at room temperature in the dark. To degrade the hydrogel and re-suspend the cells, samples were then soaked in TrypLE for 10 min and gently vortexed. The resulting solutions were aliquoted to glass slides, covered with a glass cover slip, and imaged using a Cytation 5 Multi-Mode Reader from BioTek (Winooski, VT, USA) with excitation/emission values of 494/517 nm (green channel) and 528/617 nm (red channel). Three regions from each slide were captured and percent cell viability was calculated as [# of live cells (green)/# of dead cells (red) X 100%]. Select stained samples were also imaged without construct degradation for a quick verification of channeled geometry fabrication.

For a quantitative representation of cell number and proliferation, a PrestoBlue metabolic assay (Invitrogen) was conducted on days 1 and 7 with six replicates from each group. Each sample was incubated in a 1:10 PrestoBlue-to-media solution at 37 °C for 45 min. Four 200 *µ*l aliquots were taken from each sample and pipetted into a 96-well plate. Bottom-read fluorescence intensity was measured from the 96-well plate at 560 nm excitation, 590 nm emission, and 10 nm bandwidth using a Cytation 5 Multi-Mode Reader (BioTek).

#### 2.5.5. Confocal microscopy

Monoclonal cluster of differentiation 31 (CD31) antibodies conjugated with SuperBright 436 from eBioscience (San Diego, CA, USA) and platelet-derived growth factor receptor beta (PDGFRB) antibodies conjugated with PE from Life Technologies (Carlsbad, CA, USA) were chosen to verify CD31 and PDGFRB protein expression, commonly used EC and PC markers, respectively. On day 7, six replicates from each group were washed three times with PBS and fixed with 4% (wt./vol.) paraformaldehyde for 10 min in an incubator at 37 °C and 5% CO_2_. The 4% paraformaldehyde was apirated from each sample and they were again washed three times with PBS. Each sample was then incubated with 0.1% (wt./vol.) Triton X-100 from MP Biomedicals (Santa Ana, California, USA) for 15 min to permeabilize the cell membranes. Samples were washed three more times with PBS before soaking in 2% (wt./vol.) bovine serum albumin (BSA) from Fisher Scientific (Waltham, MA, USA) at room temperature overnight to block excess protein binding sites. The 2% BSA was removed before adding the anti-CD31 and PDGFRB antibody solutions diluted in 500 *µ*l of 0.1% BSA to each well in the dark, leaving the plate in a 4 °C refrigerator covered in foil overnight. The next day, the combined antibody solution was removed and samples were washed three more times with PBS. Samples were finally stored in PBS at 4 °C covered in foil until confocal microscopy.

Samples were imaged using a 10X objective on a Nikon AXR (Minato City, Tokyo, Japan) confocal microscope, with a 405 nm excitation laser and 423–476 nm emission range for CD31, and 488 nm excitation laser and 503–660 nm emission range for PDGFRB.

### 2.6. Hydrogel rheology

All hydrogels utilized in this study were subjected to low-viscosity flow tests (i.e. frequency sweeps) on an Anton Paar (Ashland, VA, USA) MCR 102e Modular Compact Rheometer. Viscosity measurements were collected on Anton Paar’s RheoCompass software for each hydrogel between 1 1 s^−1^ and 100 1 s^−1^ shear rates.

### 2.7. Statistical analysis

Quantitative data is presented as mean *±*relative standard deviation (%). Percent error was calculated for each measured mean against a theoretical value to quantify the device’s accuracy in producing constructs with intended dimensions. Cell viability and metabolic activity data across both groups at each timepoint was analyzed for statistical significance using two-tailed, paired *t* tests, with *p*-values < 0.05 considered significant. All data was gathered in triplicate or higher.

## 3. Results and discussion

### 3.1. Verification of fabrication capabilities

#### 3.1.1. Channeled sheet production and dimensional accuracy

**Figure 5.**
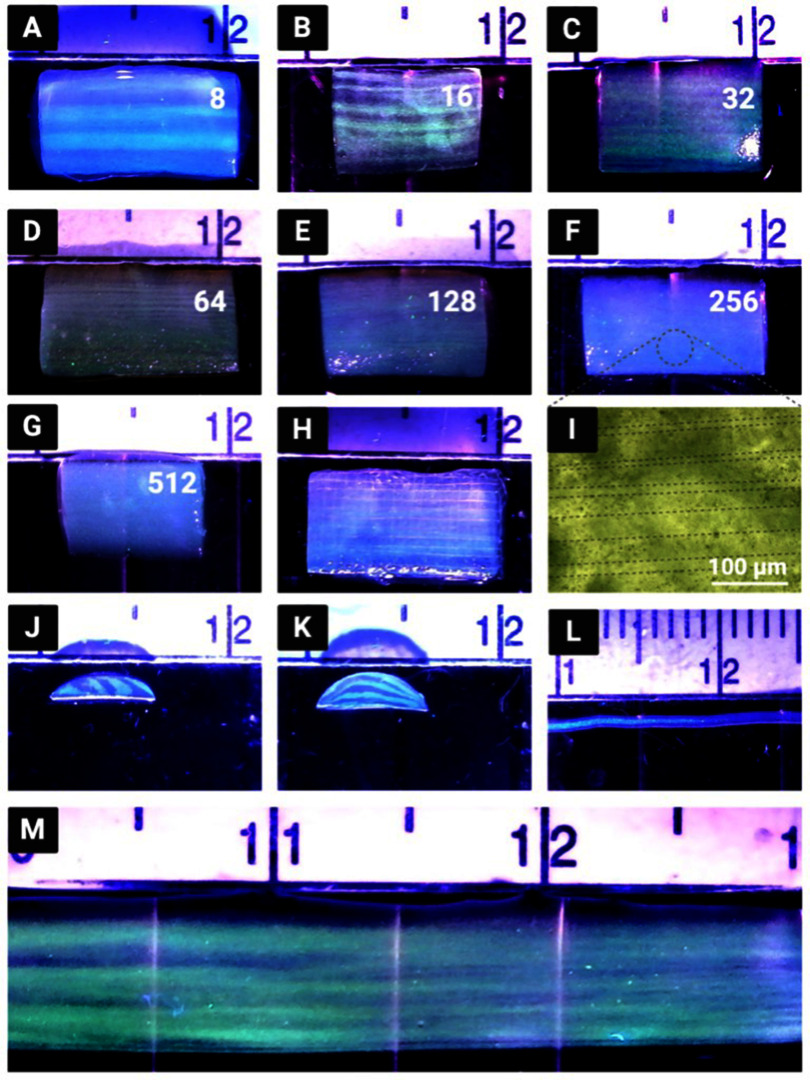
Acellular hydrogel constructs produced by the CEVIC device using fluorescent microspheres to visualize internal channels. (A)–(G) The CEVIC device can pass two hydrogel inputs through a varying number of kenics mixing elements to print sheets with 8–512 internal channels, ranging from approx. 640–10 *µ*m in width, respectively. (H) 16-channeled sheet construct fused with a melt electrowritten (MEW) scaffold, combining the benefits of two biofabrication strategies. (I) Brightfield microscope image of fluorescent microspheres, highlighting the approximately 20 *µ*m channels. (J) Cross-section of 8-channeled sheet. (K) Cross-section of 8-channeled sheet with printhead output rotated 90 degrees to produce channels parallel to the print bed. (L) CEVIC device can also print traditional filaments while switching materials within the same continuous filament. (M) CEVIC device switching between a varying number of Kenics mixing elements within a single extrusion to produce a construct with 8, to 16, and then 32 channels within one continuous sheet, mimicking the hierarchical branching structure of microvasculature. Large, medium, and small notches on the rule shown in these images depict 10, 5, and 1 mm, respectively.

The CEVIC device was shown to successfully extrude adjacent, alternating channels of two hydrogel inks within sheet geometries. Sheets were fabricated containing 8, 16, 32, 64, 128, 256, and 512 alternating internal channels, with average channel widths ranging from 621.5 ± 42.92% *µ*m to 11.67 ± 14.99% *µ*m, respectively (figures 5(A)–(G)). While the largest and smallest channel widths had the highest and lowest relative standard deviation and percent error, respectively, there was no trend in these values across all channel widths. The average percent error between measured and theoretical channel widths was calculated to be 10.74%. The measured channel widths were relatively close to their ‘theoretical’ widths (e.g. an 8-channeled 5 mm sheet should average 625 *µ*m-thick channels) (figure 7). The smallest channel width was measured as 10 *µ*m within a 512-layered sheet. It is possible that smaller channel widths could be achieved in future work. These constructs demonstrate the CEVIC device’s ability to pattern alternating channels into sheet constructs at resolutions in the range from surgically manipulatable small-diameter arteries and veins (i.e. ∼1–6 mm) to microvasculature (i.e. <100 *µ*m) down to capillaries (i.e. ∼10 *µ*m) [23, 24]. Channel orientation was also switched between vertical and horizontal by rotating the fanning outlet 90 degrees (figure 5(J) and (K)). This capability could allow for multi-cellular, multi-layered sheets of tissue (e.g. skin, organ walls) with a gradient of layer patterning to be produced with a single extrusion.

Mid-extrusion material switching from a single printhead was also demonstrated with traditional filament extrusion (figure 5(L)). Additionally, mid-extrusion pattern switching was demonstrated by transitioning from 8, to 16, then 32 channels within a continuous hydrogel sheet (figure 5(M)). This capability can allow for a single, surgically manipulatable hydrogel sheet to contain complex micropatterning reminiscent of natural tissue structures such as the hierarchically branching microvascular tree, a well-documented challenge for bioprinting [41]. The transition lengths between 8, 16, and 32-channel regions achieved with manual valve switching were approximately 1 cm. Complete automation of valve switching, as well as reducing flow rate, should allow for shorter transition lengths to be obtained. This is the subject of an ongoing study.

Sheet dimensions were measured on average to be 4.78 ± 0.07 mm wide by 1.22 ± 0.33 mm thick. Sheets tended to be thickest in the center, bloating beyond the 0.5 mm outlet thickness, while thinning to their edges. However, this variation was likely driven by wetting between the hydrogel and polystyrene well plate lid being used as a printing surface for demonstration purposes. This effect may have contributed to the 8-channel sheet having the highest variation in measured channel widths, as channels toward the middle of the construct bloated to become wider than those toward the construct edge. Sheets with more uniform thickness could be produced with more rapid crosslinking upon deposition (e.g. UV and/or CaCl_2_ exposure while printing takes place) or by using a printing surface with higher affinity for the hydrogel. Early tests of extruding sheets directly into bulk CaCl_2_ solutions produced more consistent thicknesses (not without expected elastic swelling inherent to extruding hydrogels into crosslinking solutions [42]) but were more prone to disrupted flow. This justifies the method of depositing sheets before subjecting them to curing. Other existing strategies for improving structural stability, such as gelatin embedding [43], could be integrated with the CEVIC device in future studies.

#### 3.1.2. Producing perfusable channels with fugitive ink

**Figure 6.**
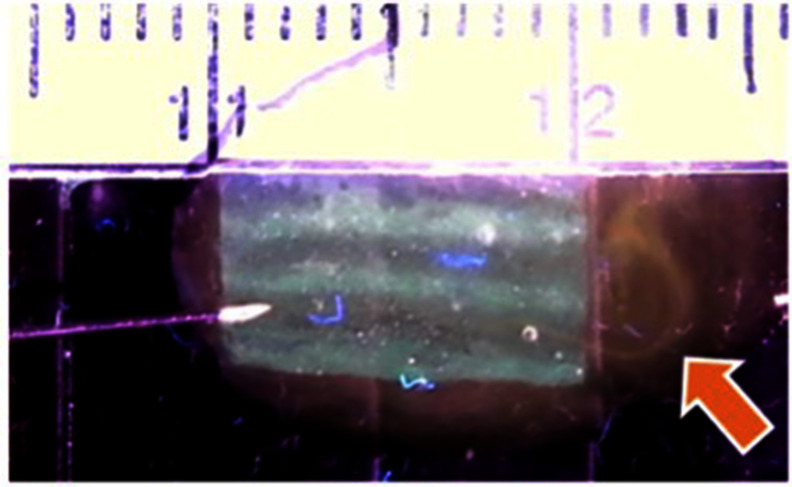
Eight-channel hydrogel sheet containing vacant channels left behind by ‘fugitive’ ink. Perfusability of a vacant channel is demonstrated by injecting it with orange-dyed water via an insulin syringe. Arrow indicates flow streamline exiting the opposite end of the sheet (small notches depict 1 mm).

Sheets were also produced with HEC fugitive ink that diffused away to leave vacant channels in between solid hydrogel channels. Orange-dyed water injected into vacant channels was able to exit the opposite end of the sheet, demonstrating their perfusability (figure 6). Not every vacant channel was shown to be perfusable with this method, likely due to channel sinking, inexact needle placement, and variable flow rate. However, even vacant channels that are not fully perfusable immediately upon fabrication could provide a framework for *in vitro* development of perfusable, endothelialized microvascular structures [44]. Further study is planned to investigate and optimize printed structures for developing prevascularized constructs.

**Figure 7.**
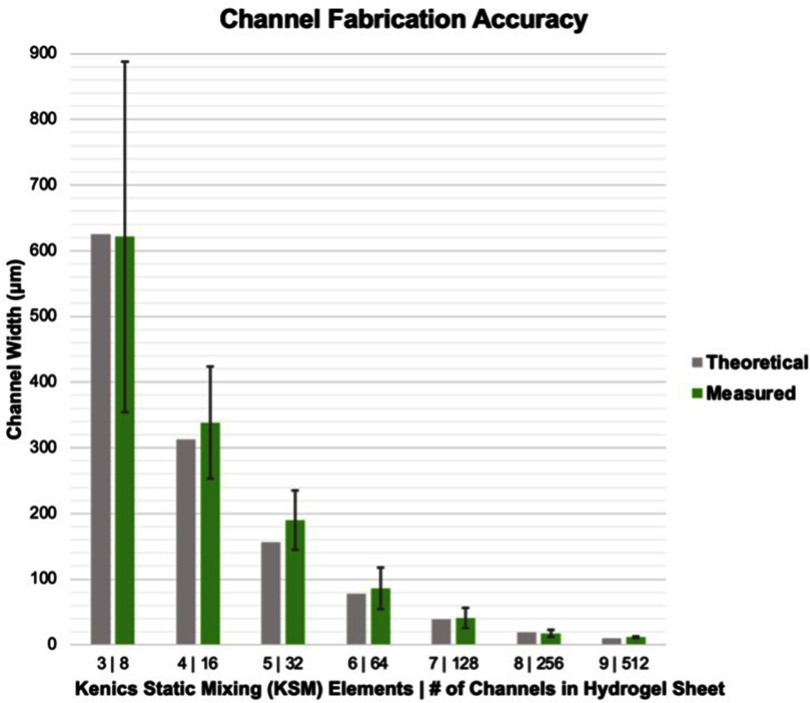
CEVIC device on average produces internal channel widths close to (i.e. within 10.74% standard error of) the intended/theoretical dimensions. Error bars represent standard deviation.

#### 3.1.3. Integration with MEW

PCL MEW scaffolds and 16-channelled hydrogel sheets were successfully fused into single constructs (figure 5(H)). While MEW has been combined with extrusion-based and inkjet bioprinting in past work [45], this is the first study combining MEW with chaotic printing, as well as with extrusion-based hydrogel sheet printing. Combining the two technologies allows for the tuned extracellular environment of hydrogels to be mechanically reinforced by the melt electrowritten woven fiber network [46]. Using the CEVIC device to deposit a channeled hydrogel sheet over a prefabricated MEW scaffold allowed the uncured hydrogel to first penetrate the MEW scaffold pores before the curing process effectively bound the two materials together. The uncured hydrogel was drawn into space within the MEW pores via capillary action between the hydrogel and PCL fibers. With larger pores (e.g. 1 mm), this disturbed enough hydrogel volume to disrupt channel structures within the deposited sheet. However, sheets deposited onto MEW scaffolds with 350 *µ*m pores or less maintained their channel patterning until curing. We are working to combine CEVIC and MEW printheads on one robotically controlled 3D printer to allow production of hybrid constructs within a single fabrication period.

#### 3.1.4. Hydrogel rheology

**Figure 8.**
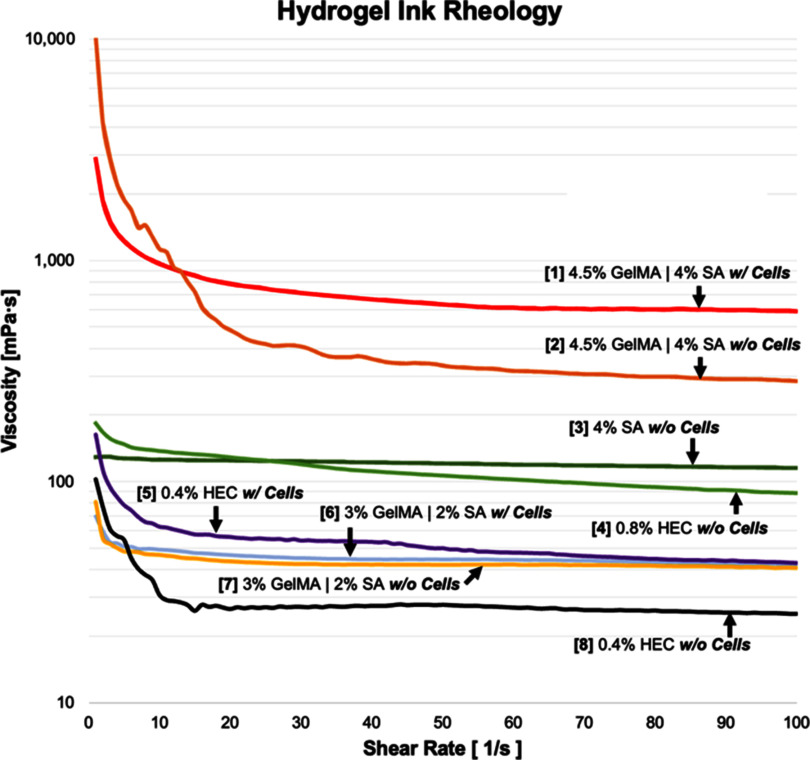
Introducing cells in GelMA-SA hydrogel formulation generally increases gel viscosity and can alter shear-thinning characteristics. Viscosities of hydrogels/inks chosen as combined inputs for the CEVIC device (i.e. #1–2, 3–4, 5–6, 7–8) in various testing scenarios can be compared with this data. Gels with similar viscosities are able to flow adjacently (i.e. not through or past each other) during channel production.

Viscosity measurements across varying shear rates for the hydrogel inks utilized in this study are presented in figure 8, providing a useful reference to compare gel viscosities while choosing materials as combined inputs for the CEVIC device. Gels inputted into the device must have relatively similar viscosities to successfully flow adjacently (i.e. not through or past each other) during channel production. Further investigations of varied concentration combinations could quantify a threshold for required viscosity ‘percent similarity’ between each device input. All inks demonstrated shear-thinning behavior, an important ink characteristic for extrusion-based bioprinting to allow flow through the printhead with minimum shear stress on cells [47]. The 4% (wt./vol.) SA and 0.8% HEC inks used for acellular testing had relatively comparable viscosities across all shear rates, justifying the choice of 0.8% as a concentration for HEC to be extruded adjacent to 4% SA to produce perfusable channels alongside SA channels. Likewise, the 3% GelMA—2% SA ink compared more closely to the 0.4% HEC fugitive ink flowed concurrently in the cell experiment. Interspersing cells into the inks raised viscosity in each tested material, but apparently not enough to disrupt the adjacent flow profiles.

### 3.2. Verifying cell viability

#### 3.2.1. Cell viability and proliferation

**Figure 9.**
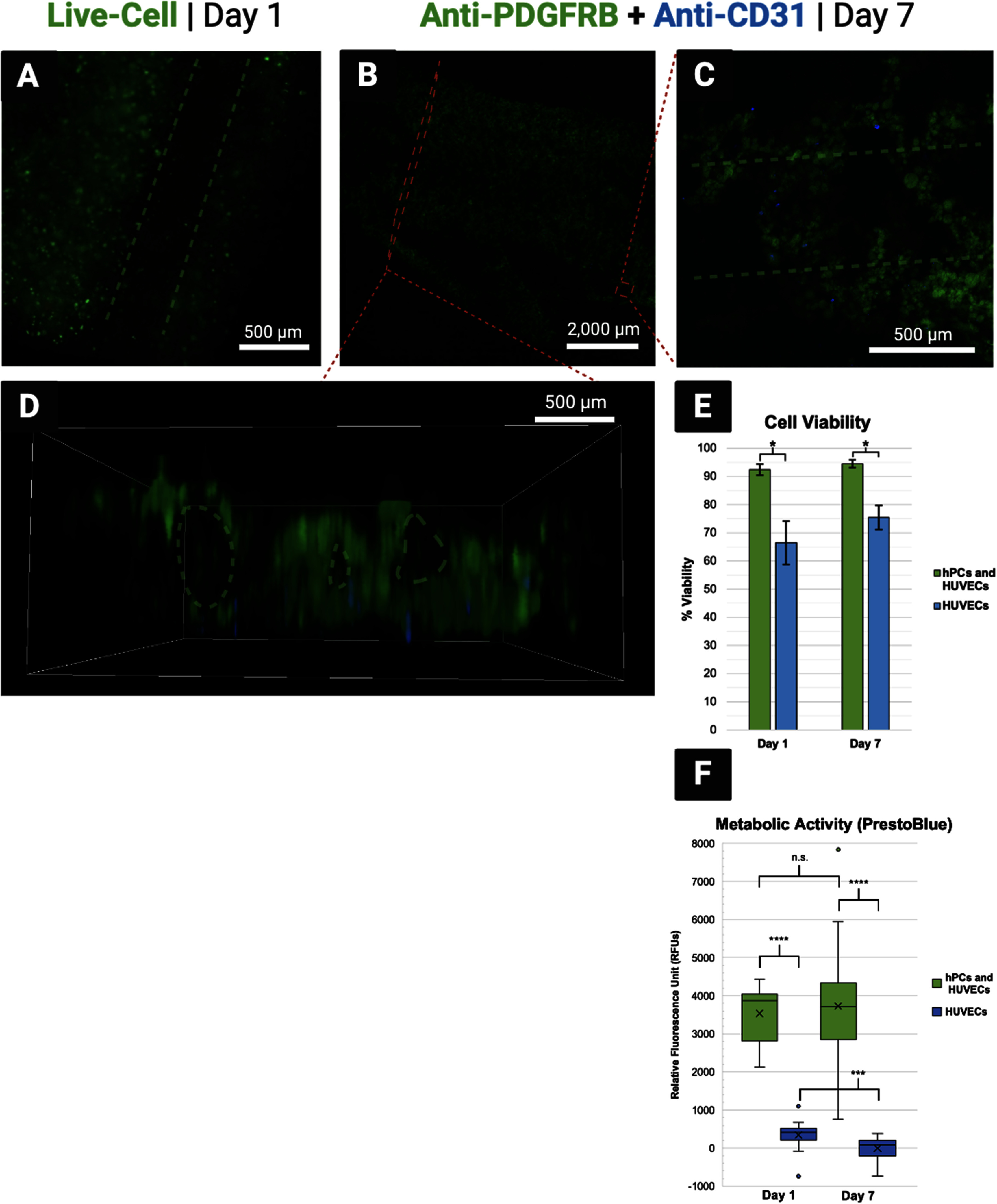
(A) Fluorescent microscopy on day 1 shows living cells illuminated green around a vacant channel. (B)–(D) Antibody tags label pericytes (hPCs) green and endothelial cells (HUVECs) blue within combined group samples. (B) An entire cell-laden sheet is visible. Cell-laden channels toward the bottom have begun to break off, likely due to 7-day hydrogel degradation accelerated by endpoint immunohistochemical processing reagents. (C) hPCs appear to have seeded with more efficiency than HUVECs, suggesting that interspersing HUVECs within the fugitive ink (‘endothelial seeding method’) before printing was inefficient compared to interspersing cells within the solidifying hydrogel ink. However, both cells are viable while positioned around a vacant channel (D) Side-view of sheet showing visibility of vacant channels that can be hindered from a top-down viewing angle. (E) Cell viability in hydrogel sheets containing both cell types was measured above 90% on both days, although viability in the hPC populations was not measured independently. Viability was lower in the HUVEC-only group, likely attributed to the apparent low-efficiency of the endothelial seeding method. (F) Relative cell numbers were significantly higher in the combined groups, suggesting again that cell numbers were significantly lower in the HUVEC populations due to the inefficient endothelial seeding method, thus contributing to lower HUVEC viability in isolation.

Average percent cell viability was measured to be 92.47 ± 0.02% and 66.43 ± 0.12% on day 1 for the experimental and control groups, respectively. On day 7, it was measured to be 94.48 ± 0.01% and 75.48 ± 0.06% for each respective group (figure 9(E)). These results indicate strong viability across the week of culture for the experimental group containing both hPCs and HUVECs, although the viability of each cell type could not be distinguished within the experimental group with the methods used. By contrast, viability was significantly lower in the HUVEC-only control group. This suggests that the endothelial seeding method attempted in this proof-of-concept was not an efficient way to seed HUVECs into the constructs. It is expected that the fugitive ink diffused out of the constructs quickly during exposure to CaCl_2_, washing most of the HUVEC population away before attachment could occur. As a result, the HUVEC populations in both groups were much lower than intended, contributing to the observably lower viability in the control group. Future efforts should attempt to seed both hPCs and HUVECs within the solid channels surrounding vacant channels as an alternative to the endothelial seeding method. To potentially improve efficiency of the endothelial seeding method, future CEVIC device studies could experiment with higher-viscosity fugitive inks, temperature-induced fugitive ink dissolution [48], or strategies that do not require CaCl_2_ immersion to cure the constructs (e.g. using UV-crosslinkable materials only). Viability would then likely be comparable between hPC and HUVEC populations. Early work seeding HUVECs alone in CEVIC-produced GelMA-SA constructs suggested strong HUVEC viability via fluorescent microscopy of calcein AM staining, although these preliminary cell viability results were not quantified (figure S1).

This theory on ineffective HUVEC seeding is supported by the significantly lower metabolic activity readings in the HUVEC-only control group on both timepoints, as compared to the hPC and HUVEC combined experimental group (figure 9(F)). While these results suggest significant combined cell populations in experimental group constructs, there was no significant increase in net metabolic activity from day 1–7 in the experimental group. This suggests either low proliferation rate or high turnover rate within the experimental group constructs, despite their relatively high percent viability.

Based on these viability results, shear stresses associated with extrusion through the CEVIC device, as well as the 30 s 365 nm UV exposure, do not appear to influence cell viability to an extent that could be detrimental to the cellular functionality of resulting constructs. However, a more in-depth analysis of the device could be performed to correlate optimal cell viability and proliferation to the maximum shear stresses upon cells (e.g. determined via finite element modeling analysis of device geometries under various hydrogel flow conditions) and UV exposure (i.e. intensity and duration).

#### 3.2.2. Fluorescent and confocal microscopy

Calcein AM staining illuminated living cells under a fluorescent microscope to demonstrate cells positioned in solid hydrogel channels around vacant channels, as intended 1 d after printing (figure 9(A)). CD31 and PDGFRB protein expression was observed from HUVECs and hPCs, respectively, in the constructs at day 7 via confocal microscopy. Both cell types are observed surviving in proximity to each other around vacant channels on day 7, despite a visibly lower number of HUVECs due to the endothelial seeding method employed (figures 9(B)–(D)).

The images shown in figure 9 are generally representative of all the samples observed, although some variations do occur across samples and between each channel. These variations are likely attributed primarily to the timespan where sheets have been printed but are not yet solidified via curing. During this time, small fluctuations can occur in the still-fluid hydrogel and fugitive inks. Additionally, sheets with vacant channels may be prone to channel collapse without sustained flow through the channels during the culture period. Fluctuations appeared to have been more substantial with the lower viscosity inks and vacant channels of the *in vitro* cell experiment, as compared to the acellular tests and preliminary *in vitro* tests of seeding HUVECs into solid channels within a higher viscosity GelMA-SA hydrogel (figure S1). These fluctuations could likely be reduced by providing instant UV and CaCl_2_ exposure upon deposition in future iterations of the CEVIC device, as well as by quickly providing media flow during *in vitro* culture.

No significant observations were made regarding changes in cell number or morphology across the 1-week culture period. However, it is likely that a longer culture period (e.g. 28 d) will be necessary to fully investigate cell behavior in CEVIC-produced constructs. Future *in vitro* experiments should investigate how to best align parameters for inducing the development of vessel-like cell structures in CEVIC-produced constructs, such as growth factor doses/combinations (in nutrient media or within the channels), cell seeding densities, hydrogel concentrations, and channel widths, as well as exploring other relevant biomaterials and cell types (e.g. mesenchymal stem cells, EC and PC sources better specified to tissue application of interest) [16]. It should be noted that promoting some of these parameters may require consideration of the impacts on others (e.g. increasing cell density could increase hydrogel viscosity to the point of producing high shear stresses during printing that decrease cell viability, thus necessitating a decrease in hydrogel concentration). Additionally, it would be relevant to run a small animal model that compares *in vivo* vascularization results with constructs that are implanted directly after printing vs constructs that are ‘pre-cultured’ *in vitro* to form functional microvasculature prior to implantation. A pre-implantation culture period is expected to be beneficial for producing a sheet construct that can form anastomoses with adjacent host vessels as quickly as possible, or even suture directly to host vessels and receive immediate perfusion (i.e. in an ideal outcome for this application). However, culturing time introduces drawbacks such as opportunity for contamination, cell senescence, and treatment delay (i.e. in the event of an eventual CEVIC-based clinical therapy). Such optimization work is well-justified by this proof-of-concept *in vitro* experiment, which demonstrates that the CEVIC device can produce viable multicellular constructs with microvascular cell types seeded around vacant microchannels.

## 4. Conclusions

To our knowledge, this study presents the first example of using extrusion-based 3D bioprinting to produce wide sheet constructs for applications other than *in situ* wound repair. Additionally, the CEVIC device successfully pairs this novel sheet extrusion method with the extremely fine channel-printing capability of chaotic printing. Chaotically printed filaments with adjacent internal channels have been shown to induce unidirectional cellular alignment in previous work. Thus, the CEVIC device can produce sheet constructs mimicking the shape of thin tissue layers for a wide variety of applications that require cellular alignment (e.g. neural, skin, muscle, bone, tendon, cartilage, ligament, cornea, vascular, etc) with precise control over the width of each cell channel. These sheet constructs can include hundreds of adjacent cell channels as thin as a single cell that are inherently fused together with one continuous extrusion. By contrast, filament-based extrusion bioprinting would require hundreds of filament passes and successful fusing between each filament to produce a construct with the same width and channel resolution. The CEVIC printhead could also, potentially, deposit unidirectional channels into a vat for subsequent light-assisted bioprinting of complex construct designs. These constructs would contain one or more cell types following an intended alignment path, bringing the capabilities of chaotic printing to light-assisted bioprinting for the first time. Future work is also planned to explore stacking (i.e. bioassembly) of CEVIC-produced hydrogel sheet constructs to obtain microvascularized 3D constructs, as well as to test directing channeled, chaotic printed constructs into new biologically relevant geometries additional to flat sheets, such as curving surfaces, hollow tubes, and solid organs.

The complex gating system of as many as 14 inputs to the CEVIC device printhead is able to start with two simple syringes driven by an air pump or syringe pump. As such, the CEVIC device could be merged with existing commercially available bioprinters and/or biofabrication devices to combine their capabilities. One such device is a MEW printer, wherein the CEVIC device was proven to produce sheet constructs that can fuse to MEW biotextile scaffolds. This could be an important step in producing composite sheet constructs with enhanced material strength that can be handled and sutured as an implant during future *in vivo* studies. Additionally, the CEVIC device printhead was shown to be operable by hand, providing the potential for *in situ* bioprinting applications, such as depositing a multi-material skin graft containing aligned cells directly over a wound site.

This study also presents a novel method of switching bioink inputs and/or micropatterning mid-extrusion that can either be controlled manually via mechanical valves or fully automated via electronically controlled valves as part of a printing sequence. The CEVIC device can switch the number and width of channels mid-extrusion, from the mm-scale of structures such as small-diameter blood vessels to the 10 *µ*m-scale of capillaries, in continuous succession. The CEVIC device can thus produce a hiearchically branching network of microvasculature-like channels across length scales not yet achieved in other microvascular fabrication strategies.

This study proved the viability and continued vascular expression of ECs and PCs co-seeded in sheet constructs for at least a week, suggesting the potential of the CEVIC device for developing microvascular tissue constructs. Furthermore, the use of a fugitive ink has been demonstrated to create perfusable vacant channels that could potentially guide development of functional microvasculature in cell-seeded sheet constructs. These constructs could eventually be applied as implants to promote vascularization in wound sites, microvasculature supply to larger tissue grafts or biofabricated graft constructs, or as tissue models for drug screening and disease modeling.

However, further investigation is required to advance from the current state of maintaining viable microvascular cells within these constructs to the desired outcome of functional microvascular tissue. *In vitro* research is ongoing to provide more in-depth analysis of microvascular cell morphology and function within CEVIC-produced constructs. This work is expected to inform optimization of pre-culture and/or implantation parameters resulting in functional, perfusable microvascular sheet implants for a small animal study, as the first application utilizing the CEVIC device presented here.

## Data Availability

All data that support the findings of this study are included within the article (and any supplementary files).
